# Racial, Ethnic, and Socioeconomic Differences in Food Allergies in the US

**DOI:** 10.1001/jamanetworkopen.2023.18162

**Published:** 2023-06-14

**Authors:** Jialing Jiang, Christopher M. Warren, Audrey Brewer, Gary Soffer, Ruchi S. Gupta

**Affiliations:** 1 Center for Food Allergy & Asthma Research, Northwestern University Feinberg School of Medicine, Chicago, Illinois; 2 Advanced General Pediatrics and Primary Care, Ann & Robert H. Lurie Children’s Hospital of Chicago, Chicago, Illinois; 3 Department of Pediatric Pulmonology, Allergy, Immunology and Sleep Medicine, Yale School of Medicine, New Haven, Connecticut

## Abstract

**Importance:**

Food allergies affect approximately 8% of children and 11% of adults in the US. Racial differences in food allergy outcomes have previously been explored among Black and White children, but little is known about the distribution of food allergies across other racial, ethnic, and socioeconomic subpopulations.

**Objective:**

To estimate the national distribution of food allergies across racial, ethnic, and socioeconomic groups in the US.

**Design, Setting, and Participants:**

In this cross-sectional survey study, conducted from October 9, 2015, to September 18, 2016, a population-based survey was administered online and via telephone. A US nationally representative sample was surveyed. Participants were recruited using both probability- and nonprobability-based survey panels. Statistical analysis was performed from September 1, 2022, through April 10, 2023.

**Exposures:**

Demographic and food allergy–related participant characteristics.

**Main Outcomes and Measures:**

Stringent symptom criteria were developed to distinguish respondents with a “convincing” food allergy from those with similar symptom presentations (ie, food intolerance or oral allergy syndrome), with or without physician diagnosis. The prevalence of food allergies and their clinical outcomes, such as emergency department visits, epinephrine autoinjector use, and severe reactions, were measured across race (Asian, Black, White, and >1 race or other race), ethnicity (Hispanic and non-Hispanic), and household income. Complex survey-weighted proportions were used to estimate prevalence rates.

**Results:**

The survey was administered to 51 819 households comprising 78 851 individuals (40 443 adults and parents of 38 408 children; 51.1% women [95% CI, 50.5%-51.6%]; mean [SD] age of adults, 46.8 [24.0] years; mean [SD] age of children, 8.7 [5.2] years): 3.7% Asian individuals, 12.0% Black individuals, 17.4% Hispanic individuals, 62.2% White individuals, and 4.7% individuals of more than 1 race or other race. Non-Hispanic White individuals across all ages had the lowest rate of self-reported or parent-reported food allergies (9.5% [95% CI, 9.2%-9.9%]) compared with Asian (10.5% [95% CI, 9.1%-12.0%]), Hispanic (10.6% [95% CI, 9.7%-11.5%]), and non-Hispanic Black (10.6% [95% CI, 9.8%-11.5%]) individuals. The prevalence of common food allergens varied by race and ethnicity. Non-Hispanic Black individuals were most likely to report allergies to multiple foods (50.6% [95% CI, 46.1%-55.1%]). Asian and non-Hispanic White individuals had the lowest rates of severe food allergy reactions (Asian individuals, 46.9% [95% CI, 39.8%-54.1%] and non-Hispanic White individuals, 47.8% [95% CI, 45.9%-49.7%]) compared with individuals of other races and ethnicities. The prevalence of self-reported or parent-reported food allergies was lowest within households earning more than $150 000 per year (8.3% [95% CI, 7.4%-9.2%]).

**Conclusions and Relevance:**

This survey study of a US nationally representative sample suggests that the prevalence of food allergies was highest among Asian, Hispanic, and non-Hispanic Black individuals compared with non-Hispanic White individuals in the US. Further assessment of socioeconomic factors and corresponding environmental exposures may better explain the causes of food allergy and inform targeted management and interventions to reduce the burden of food allergies and disparities in outcomes.

## Key Points

**Question:**

What is the national distribution of food allergies among all US individuals across race, ethnicity, and socioeconomic groups?

**Findings:**

In this survey study of 51 819 households, Asian, Black, and Hispanic individuals were more likely to report having food allergies compared with White individuals. The prevalence of food allergies was lowest among households in the highest income bracket.

**Meaning:**

This study suggests that racial, ethnic, and socioeconomic differences in the prevalence of food allergies exist and are evident in clinical outcomes such as food allergy–related emergency department visits and epinephrine autoinjector use.

## Teaser

This survey study of a nationally representative US sample estimates the distribution of food allergies across racial, ethnic, and socioeconomic groups.

## Introduction

Food allergies (FAs) affect an estimated 8% of children and 11% of adults in the US.^1,2^ Individuals with an FA may experience FA-related economic burden, lower health-related quality of life, and increased risk of comorbid atopic conditions (ie, eczema, asthma, and/or allergic rhinitis).^3^ However, the distribution of FA burden may vary across different racial, ethnic, and socioeconomic strata.^4,5^

The prevalence of self-reported FAs has been increasing in recent decades, especially among non-Hispanic Black (hereafter, Black) children.^6^ Black children have been reported to have higher rates of FAs compared with non-Hispanic White (hereafter, White) children in the US.^7,8^ In the 2007-2010 National Health and Nutrition Examination Survey (NHANES), 8.1% of Black children had parent-reported FAs compared with 6.3% of White children and 5.2% of Hispanic children.^9^ Black children also often had higher food-specific immunoglobulin E (IgE) levels.^10,11,12^ In a Boston-area birth cohort study, Black children were reported to be more likely to be sensitized to any food allergens and multiple food allergens compared with White children.^13^ Less is known about racial differences in FAs among adults, although the limited available evidence suggests that the differences reported in pediatric samples may also exist among adults.^14^ The NHANES sensitization data from 2005-2006 suggested that serologically defined FA to peanut, egg white, cow’s milk, and shrimp was more common among Black children and adults.^12^ These study findings and others, compiled using medical record review and random digit dial survey methods, concluded that Black children and adults have higher rates of seafood allergy compared with other races and ethnicities.^4,15,16^

Despite a growing body of literature on racial differences in FA prevalence and phenotypes between Black and White populations, there remains a paucity of population-based data on FA burden among other races and ethnicities in the US across all age groups—particularly within the past decade. In addition, although a complex interplay between race and socioeconomic factors exists, these social determinants of health remain underexplored in FA research, to our knowledge.^5^ Therefore, this study aimed to estimate the distribution of self-reported or parent-reported, “convincing” FAs, reaction severity, and management among individuals of varying racial, ethnic, and socioeconomic backgrounds in the US.

## Methods

Between October 9, 2015, and September 18, 2016, a population-based survey was developed and administered to 51 819 US households, obtaining parent-reported responses for 38 408 children (≤18 years) and self-reported responses from 40 443 adults (>18 years). Adults completed the survey in English or Spanish via telephone or online. The probability-based sampling methods used included additional coverage of rural and low-income households that are frequently underrepresented in surveys relying on address-based or convenience sampling.^1,2^ The institutional review boards of Northwestern University and NORC (National Opinion Research Center) at the University of Chicago approved all research study activities. Written and oral informed consent was obtained from all participants. This study followed the American Association for Public Opinion Research (AAPOR) reporting guidelines.

### Outcome Measures

Primary outcome measures included overall pediatric and adult self-reported prevalence of any FA(s) to 9 common, federally regulated food allergens (cow’s milk, hen’s egg, peanut, tree nuts, soy, wheat, sesame, fin fish, and shellfish) among various racial and ethnic groups. Data on physician-diagnosed comorbid atopic conditions, allergic reaction symptoms, severe FAs, emergency department (ED) visits, epinephrine prescriptions, and presence of multiple FAs were also obtained.

Self-reported or parent-reported FA prevalence was calculated for physician-confirmed FAs and “convincing” FAs (self-reported or parent-reported FAs corroborated by a history of symptoms related to an IgE-mediated FA). Self-reported or parent-reported convincing FAs were identified using a stringent algorithm that incorporated a stringent IgE-mediated FA symptom list and reported food allergens. The algorithm was designed to exclude reported FA cases that did not have a clinical food-specific reaction history indicative of a true IgE-mediated FA, such as suspected food intolerances and oral allergy syndrome.^1,2^ “Physician-confirmed FAs” (hereafter, confirmed FAs) met the criteria for convincing FAs but were also reported as physician diagnosed via confirmatory oral food challenge, skin prick, and/or specific IgE testing. Food allergies were considered severe if stringently defined symptoms were reported that involved 2 or more organ systems as defined: (1) skin and/or oral mucosa system: hives, swelling, lip and/or tongue swelling, difficulty swallowing, or throat tightening; (2) respiratory system: chest tightening, trouble breathing, or wheezing; (3) gastrointestinal system: vomiting; and (4) cardiovascular and/or heart system: chest pain, rapid heart rate, fainting, dizziness, feeling lightheaded, or low blood pressure.^1,2^

### Assessment of Race, Ethnicity, and Socioeconomic Status

Race is a sociopolitically constructed categorization based on phenotypic indicators. Ethnicity is also a distinct social construct that refers to a shared cultural origin.^17^ US Census definitions were used for race (ie, American Indian or Alaska Native, Asian, Black or African American, Native Hawaiian or Other Pacific Islander, White, >1 race, or other) and ethnicity (ie, Hispanic or Latino and not Hispanic or Latino).^18^ Due to sample size limitations, Native Hawaiian or Other Pacific Islander, American Indian or Alaska Native, and those who reported more than 1 race or other race were collapsed into a “more than 1 or other race” category. Therefore, presented estimates are stratified across the following 5 racial and ethnic categories: Asian, Black, Hispanic, White, and more than 1 or other race.

The socioeconomic factors assessed by the survey included household income (<$25 000, $25 000-$49 999, $50 000-$99 999, $100 000-$149 999, or ≥$150 000) and insurance type (uninsured, private insurance, or public insurance), all of which were self-reported. Insurance status was available only for a subset of 6761 AmeriSpeak panelists.

### Statistical Analysis

Statistical analysis was performed from September 1, 2022, through April 10, 2023. Self-reported confirmed and convincing FA prevalence estimates were calculated using complex survey weighted proportions.^1,2^ Pearson χ^2^ statistics were calculated to test the independence of key study variables. Covariate-adjusted, complex survey-weighted logistic regression models compared relative prevalence and other convincing FA outcomes by participant characteristics, including interaction terms to assess moderation effects by demographic information. Two-sided hypothesis tests were used, and conventional thresholds of *P* < .05 denoted statistical significance. Stata MP, version 16 (StataCorp LLC), was used for all analyses.

## Results

### Demographic Characteristics and Convincing FA Prevalence

Surveys were completed for 78 851 individuals (self-reported for 40 443 adults and parents or proxies for 38 408 children; 51.1% women [95% CI, 50.5%-51.6%]; mean [SD] age of adults, 46.8 [24.0] years; mean [SD] age of children, 8.7 [5.2] years). Table 1 presents demographic characteristics of all respondents and those with convincing FA, separately. The sample comprised 3.7% Asian individuals, 12.0% Black individuals, 17.4% Hispanic individuals, 62.2% White individuals, and 4.7% individuals of more than 1 race or other race. Observed weighted distributions by age, sex, race, and ethnicity were comparable to the general US population.^18^

**Table 1.** Demographic Characteristics of All Children and Adults Surveyed and Those With an FA

Characteristic	All US children and adults (n = 78 851)		Children and adults with convincing FA (n = 9726)	
	No.^a^	% (95% CI)	No.^a^	% (95% CI)
Race and ethnicity				
Asian	3119^b^	3.7 (3.5-4.0)^b^	410^b^	3.9 (3.4-4.4)^b^
Black, non-Hispanic	7687^b^	12 (11.6-12.5)^b^	1024^b^	12.7 (11.6-13.9)^b^
Hispanic	8636^b^	17.4 (16.7-18.1)^b^	1368^b^	18.3 (16.9-19.7)^b^
White, non-Hispanic	54 990	62.2 (61.4-62.9)	6326	58.9 (57.3-60.6)
Multiple or other^c^	4439^b^	4.7 (4.4-4.9)^b^	598^b^	6.2 (5.4-7.2)^b^
Sex				
Female	41 29 ^b^	51.1 (50.5-51.6)^b^	5439^b^	63.3 (61.9-64.8)^b^
Male	37 573^b^	48.9 (48.4-49.5)^b^	4287^b^	36.7 (35.2-38.1)^b^
Age, y				
<1	1851^b^	1.2 (1.1-1.3)^b^	92^b^	0.3 (0.3-0.4)^b^
1	1817^b^	1.1 (1.0-1.2)^b^	166^b^	1.0 (0.7-1.3)^b^
2	2102^b^	1.3 (1.2-1.4)^b^	194^b^	1.3 (0.9-1.8)^b^
3-5	6164^b^	3.6 (3.5-3.8)^b^	550^b^	3.0 (2.6-3.6)^b^
6-10	10 524^b^	6.2 (6.0-6.5)^b^	967^b^	5.0 (4.4-5.5)^b^
11-13	6663^b^	3.7 (3.5-3.9)^b^	631^b^	2.8 (2.4-3.3)^b^
14-17	9295^b^	5.2 (5.0-5.5)^b^	832^b^	3.7 (3.3-4.1)^b^
18-29	8336^b^	16.7 (16.2-17.2)^b^	1593^b^	18.7 (17.5-20.0)^b^
30-39	7803^b^	13.2 (12.8-13.5)^b^	1446^b^	16.6 (15.5-17.8)^b^
40-49	6289^b^	13.0 (12.6-13.4)^b^	1002^b^	13.0 (12.0-14.1)^b^
50-59	7799^b^	14.0 (13.6-14.4)^b^	1062^b^	16.5 (15.4-17.8)^b^
≥60	10 218^b^	20.8 (20.3-21.3)^b^	1189^b^	18.1 (16.9-19.4)^b^
Annual household income, $				
<25 000	12 943^b^	16.5 (16.0-17.0)^b^	1554^b^	16.2 (15.1-17.4)^b^
25 000-49 999	19 653^b^	22.0 (21.4-22.6)^b^	2465^b^	22.4 (21.2-23.7)^b^
50 000-99 999	28 537^b^	31.0 (30.3-31.7)^b^	3651^b^	33.0 (31.6-34.6)^b^
100 000-149 999	11 635^b^	19.5 (18.9-20.2)^b^	1374^b^	19.3 (17.9-20.8)^b^
≥150 000	6103^b^	11.0 (10.5-11.6)^b^	682^b^	9.0 (8.1-10.1)^b^
Insurance status				
Uninsured	525	8.0 (6.7-9.6)	56	8.4 (5.8-12.0)
Private insurance	4310	66.3 (64.1-68.5)	429	63.0 (57.9-67.9)
Public insurance	1926	25.6 (23.8-27.6)	219	28.6 (24.2-33.4)
Geographic region				
West	16 872	23.8 (23.1-24.5)	2227	24.7 (23.3-26.1)
Midwest	17 983	20.9 (20.3-21.4)	2015	19.9 (18.7-21.1)
South	29 292	37.7 (36.9-38.4)	3620	36.8 (35.3-38.4)
Northeast	14 328	17.7 (17.1-18.3)	1827	18.7 (17.3-20.1)
Physician-diagnosed comorbid conditions				
Asthma	9510^b^	12.2 (11.8-12.7)^b^	2549^b^	25.3 (23.9-26.7)^b^
Atopic dermatitis or eczema	4718^b^	6.5 (6.2-6.9)^b^	1156^b^	12.5 (11.5-13.6)^b^
Eosinophilic esophagitis	163^b^	0.2 (0.1-0.2)^b^	74^b^	0.6 (0.5-0.9)^b^
Food protein–induced enterocolitis syndrome	374^b^	0.3 (0.2-0.3)^b^	240^b^	1.5 (1.2-1.8)^b^
Allergic rhinitis	14 164^b^	19.5 (19.0-20.0)^b^	3094^b^	33.6 (32.2-35.2)^b^
Insect sting allergy	2443^b^	3.5 (3.3-3.7)^b^	739^b^	7.8 (7.1-8.7)^b^
Latex allergy	1534^b^	2.0 (1.9-2.2)^b^	593^b^	6.2 (5.5-7.0)^b^
Medication allergy	7269^b^	11.3 (11.0-11.7)^b^	1699^b^	20.8 (19.6-22.2)^b^
Urticaria or chronic hives	587^b^	0.8 (0.7-0.9)^b^	215^b^	2.2 (1.8-2.6)^b^
Other chronic condition	4285^b^	6.4 (6.1-6.7)^b^	755^b^	8.4 (7.6-9.3)^b^

Abbreviation: FA, food allergy.

^a^ Sample size (No.) presented is unweighted. Point estimates are weighted to reflect the national population.

^b^ Significant at *P* < .05.

^c^ Other race category includes those who self-reported their race as other, American Indian or Alaska Native, or Native Hawaiian or Other Pacific Islander.

An estimated 5.0% of individuals in the US, across all age groups, have a physician-confirmed FA, while 10.1% have a convincing FA. By observing confirmed and convincing FA rates by age, this study found that FA rates increased during pediatric ages, plateaued during adulthood, and decreased during geriatric years among all race and ethnicity categories (eFigure in Supplement 1).

By comparing rates of convincing FAs by race and ethnicity, this study found that 10.5% (95% CI, 9.1%-12.0%) of Asian individuals, 10.6% (95% CI, 9.8%-11.5%) of Black individuals, 10.6% (95% CI, 9.7%-11.5%) of Hispanic individuals, and 9.5% of (95% CI, 9.2%-9.9%) White individuals had a convincing FA (Table 2). Black children had the highest rate of convincing FAs (8.9% [95% CI, 7.6%-10.3%]), and Asian children had the lowest rate at 6.5% (95% CI, 5.1%-8.2%) (Table 3). Black children had the highest rate of convincing peanut allergy (3.0% [95% CI, 2.4%-3.8%]) compared with all other race and ethnicity categories. Asian children reported higher rates of tree nut allergy compared with children from other racial and ethnic groups (2.0% [95% CI, 1.2%-3.2%]). Black children reported the highest rate of egg allergy (1.6% [95% CI, 1.0%-2.7%]) and fin fish allergy (0.9% [95% CI, 0.6%-1.5%]). Among the adult population, White adults had the lowest rate of convincing FAs (10.1% [95% CI, 9.7%-10.6%]) compared with other races and ethnicities, which were comparable in rates. The prevalence of peanut allergy (2.9% [95% CI, 2.0%-4.2%]) and the prevalence of shellfish allergy (3.8% [95% CI, 3.0%-4.9%]) were highest among Asian adults. Tree nut allergy prevalence was highest among Black adults (1.6% [95% CI, 1.2%-2.1%]). Hen’s egg allergy prevalence (1.2% [95% CI, 0.8%-1.8%]) and fin fish allergy prevalence (1.5% [95% CI, 1.1%-1.9%]) were highest among Hispanic adults.

**Table 2.** Prevalence of Convincing Food Allergies and Specific Food Allergens

Characteristic	All		Peanut		Milk		Shellfish		Tree nut		Egg		Fin fish		Wheat		Soy		Sesame	
	No.^a^	% (95% CI)	No.^a^	% (95% CI)	No.^a^	% (95% CI)	No.^a^	% (95% CI)	No.^a^	% (95% CI)	No.^a^	% (95% CI)	No.^a^	% (95% CI)	No.^a^	% (95% CI)	No.^a^	% (95% CI)	No.^a^	% (95% CI)
**Race and ethnicity**																				
Asian	410	10.5 (9.1-12.0)	122	2.9 (2.1-3.9)	76	1.5 (1.1-2.0)	129	3.4 (2.7-4.3)	58	1.3 (0.9-1.8)	52	1.1 (0.8-1.6)	37	0.9 (0.6-1.3)	13	0.4 (0.2-0.7)	28	0.7 (0.4-1.1)	7	0.2 (0.1-0.4)
Black	1024	10.6 (9.8-11.5)	282	2.5 (2.1-3.0)	212	2.3 (1.9-2.8)	303	3.0 (2.6-3.0)	166	1.6 (1.3-2.0)	116	1.2 (0.9-1.6)	95	0.9 (0.7-1.1)	51	0.6 (0.4-0.8)	60	0.5 (0.4-0.7)	30	0.2 (0.2-0.4)
Hispanic or Latino	1368	10.6 (9.7-11.5)	398	2.4 (2.1-2.8)	289	2.5 (2.0-3.0)	366	2.8 (2.4-3.3)	209	1.4 (1.2-1.7)	135	1.1 (0.8-1.5)	139	1.2 (1.0-1.6)	84	0.7 (0.5-0.9)	97	0.8 (0.6-1.1)	46	0.3 (0.2-0.5)
White	6326	9.5 (9.2-9.9)	1357	1.6 (1.4-1.7)	1243	1.6 (1.5-1.8)	1406	2.3 (2.1-2.5)	825	1.1 (1.0-1.2)	545	0.6 (0.6-0.7)	477	0.7 (0.6-0.7)	459	0.8 (0.7-0.9)	361	0.5 (0.4-0.6)	159	0.2 (0.2-0.3)
Multiple or other^b^	598	13.4 (11.8-15.3)	126	1.6 (1.2-2.1)	142	3.0 (2.2-4.2)	127	3.3 (2.4-4.4)	73	1.0 (0.7-1.4)	60	1.0 (0.7-1.4)	40	0.8 (0.5-1.3)	39	0.6 (0.4-0.9)	34	0.8 (0.5-1.3)	9	0.2 (0.1-0.4)
χ^2^ Value	7.8		85.5		79.4		41.3		26.2		53		49.1		16		21		8.1	
* P* value^c^	<.001		<.001		<.001		.001		.002		<.001		<.001		<.05		.01		.17	
**Household income, $**																				
<25 000	1554	9.9 (9.2-10.6)	275	1.5 (1.2-1.7)	326	1.9 (1.7-2.3)	395	2.6 (2.2-3.0)	177	1.1 (0.9-1.4)	133	0.8 (0.6-1.1)	164	1.1 (0.9-1.4)	97	0.7 (0.5-0.9)	106	0.8 (0.6-1.0)	26	0.2 (0.1-0.3)
25 000 to 49 999	2465	10.3 (9.7-10.8)	511	1.7 (1.5-1.9)	499	2.0 (1.8-2.3)	555	2.4 (2.1-2.7)	318	1.2 (1.0-1.4)	208	0.8 (0.6-0.9)	176	0.7 (0.6-0.9)	153	0.7 (0.5-0.9)	141	0.7 (0.5-0.8)	49	0.2 (0.2-0.3)
50 000 to 99 999	3651	10.7 (10.2-11.3)	944	2.2 (2.0-2.4)	781	2.1 (1.9-2.4)	910	2.8 (2.5-3.1)	507	1.3 (1.1-1.5)	365	0.9 (0.8-1.1)	291	0.8 (0.7-0.9)	248	0.8 (0.6-0.9)	219	0.6 (0.5-0.8)	115	0.3 (0.2-0.3)
100 000 to 149 999	1374	10.0 (9.2-10.8)	383	2.2 (1.9-2.6)	239	1.7 (1.3-2.1)	311	2.6 (2.2-3.0)	215	1.3 (1.0-1.5)	149	0.9 (0.7-1.3)	103	0.7 (0.5-0.9)	100	0.8 (0.6-1.1)	74	0.4 (0.3-0.6)	39	0.3 (0.2-0.4)
≥150 000	682	8.3 (7.4-9.2)	172	1.6 (1.3-2.0)	117	1.6 (1.2-2.2)	160	2.0 (1.6-2.5)	114	1.0 (0.8-1.3)	53	0.4 (0.3-0.6)	54	0.6 (0.4-0.9)	48	0.6 (0.4-0.8)	40	0.4 (0.3-0.6)	22	0.2 (0.1-0.3)
χ^2^ Value	5.1		38.4		14.6		17.3		5.7		26.9		25.8		5.4		23.8		5.7	
* P* value^c^	<.001		<.001		.22		.09		.44		.01		.004		.63		.008		.41	
**Insurance status**																				
Uninsured	56	10.3 (7.4-14.3)	4	0.3 (0.1-1.0)	14	2.0 (1.1-3.8)	14	2.4 (1.3-4.7)	6	0.6 (0.3-1.6)	2	0.8 (0.2-3.4)	5	1.5 (0.5-4.0)	1	0.1 (0.0-0.6)	4	0.7 (0.2-2.5)	0	0
Private insurance	429	9.4 (8.3-10.5)	62	1.2 (0.9-1.7)	81	2.0 (1.4-2.6)	93	2.2 (1.7-2.9)	55	1.1 (0.8-1.5)	26	0.4 (0.3-0.6)	18	0.4 (0.2-0.6)	38	0.9 (0.6-1.3)	23	0.5 (0.3-0.9)	6	0.2 (0.1-0.5)
Public insurance	219	11.0 (9.3-12.9)	13	0.8 (0.4-1.7)	45	2.2 (1.5-3.2)	51	2.6 (1.9-3.7)	16	0.7 (0.3-1.5)	12	0.7 (0.3-1.4)	19	0.9 (0.5-1.4)	18	0.9 (0.4-1.8)	8	0.5 (0.2-1.0)	3	0.1 (0.0-0.3)
χ^2^ Value	1.2		5.7		0.45		0.7		3.1		2.4		1.37		4.2		0.6		1.6	
* P* value^c^	.32		.13		.85		.78		.28		.52		.01		.18		.83		.52	

^a^ Sample size (No.) presented is unweighted. Point estimates are weighted to reflect the national population.

^b^ Other race category includes those who self-reported their race as other, American Indian or Alaska Native, or Native Hawaiian or Other Pacific Islander.

^c^ Significant at *P* < .05.

**Table 3.** Prevalence of Convincing Food Allergies According to Race, Ethnicity, Income, and Insurance

Characteristic	All		Peanut		Milk		Shellfish		Tree nut		Egg		Fin fish		Wheat		Soy		Sesame	
	No.^a^	% (95% CI)	No.^a^	% (95% CI)	No.^a^	% (95% CI)	No.^a^	% (95% CI)	No.^a^	% (95% CI)	No.^a^	% (95% CI)	No.^a^	% (95% CI)	No.^a^	% (95% CI)	No.^a^	% (95% CI)	No.^a^	% (95% CI)
**Race and ethnicity in pediatric population only**																				
Asian	116	6.5 (5.1-8.2)	47	2.6 (1.8-3.8)	28	1.6 (1.0-2.7)	30	1.7 (1.1-2.7)	26	2.0 (1.2-3.2)	16	1.0 (0.5-1.9)	9	0.5 (0.2-1.2)	3	0.3 (0.1-1.4)	6	0.4 (0.1-1.3)	3	0.3 (0.1-1.4)
Black	261	8.9 (7.6-10.3)	138	3.0 (2.4-3.8)	75	2.2 (1.5-3.3)	98	2.2 (1.7-2.9)	73	1.6 (1.1-2.2)	55	1.6 (1.0-2.7)	40	0.9 (0.6-1.5)	18	0.4 (0.2-0.7)	22	0.6 (0.3-1.1)	14	0.3 (0.2-0.6)
Hispanic or Latino	493	8.4 (7.2-9.8)	168	2.5 (1.9-3.2)	116	2.2 (1.5-3.1)	92	1.5 (0.8-2.0)	89	1.3 (1.0-1.7)	55	0.9 (0.6-1.2)	30	0.8 (0.5-1.3)	24	0.4 (0.3-0.7)	30	0.5 (0.3-0.8)	17	0.3 (0.2-0.5)
White	2186	7.0 (6.4-7.6)	662	1.8 (1.6-2.1)	547	1.8 (1.6-2.1)	329	1.0 (0.8-1.2)	352	1.0 (0.8-1.2)	252	0.7 (0.7-0.9)	141	0.4 (0.3-0.5)	140	0.6 (0.4-0.8)	133	0.4 (0.3-0.7)	64	0.2 (0.1-0.2)
Multiple or other^b^	276	8.1 (6.7-9.8)	86	2.5 (1.8-3.6)	71	1.7 (1.3-2.3)	50	1.1 (0.8-1.5)	47	1.3 (0.9-2.1)	40	1.3 (0.8-2.3)	17	0.4 (0.2-0.6)	20	0.5 (0.3-0.8)	13	0.4 (0.2-0.8)	4	0.1 (0.0-0.2)
χ^2^ Value	3.2		65.3		14.7		114.1		42.5		87.1		67.7		9.6		4.5		22.8	
*P* value^c^	.02		.01		.64		<.001		.03		.007		.008		.67		.91		.16	
**Annual household income in pediatric population only, $**																				
<25 000	380	7.3 (6.1-8.6)	104	1.7 (1.3-2.3)	99	1.8 (1.2-2.7)	66	1.3 (0.8-2.0)	51	1.0 (0.6-1.6)	45	0.7 (0.4-1.3)	38	1.0 (0.6-1.8)	15	0.3 (0.1-0.6)	28	0.6 (0.3-1.3)	7	0.1 (0.0-0.4)
25 000 to 49 999	771	8.0 (7.1-9.0)	210	1.8 (1.5-2.3)	195	2.2 (1.7-2.8)	104	1.1 (0.9-1.5)	114	1.0 (0.8-1.3)	82	0.8 (0.6-1.2)	41	0.4 (0.3-0.7)	43	0.6 (0.3-1.0)	50	0.7 (0.4-1.1)	19	0.2 (0.1-0.4)
50 000 to 99 999	1408	7.7 (7.0-8.4)	464	2.3 (2.0-2.7)	365	2.1 (1.7-2.6)	275	1.4 (1.2-1.7)	247	1.3 (1.1-1.6)	174	1.1 (0.8-1.6)	102	0.6 (0.4-0.8)	92	0.5 (0.4-0.8)	79	0.4 (0.3-0.6)	49	0.2 (0.2-0.4)
100 000 to 149 999	582	8.1 (6.8-9.6)	217	2.7 (2.1-3.6)	127	2.0 (1.3-3.1)	94	1.1 (0.8-1.4)	110	1.4 (1.1-1.8)	86	1.0 (0.8-1.3)	32	0.3 (0.2-0.5)	36	0.6 (0.3-1.0)	30	0.3 (0.2-0.5)	15	0.2 (0.1-0.4)
≥150 000	291	6.4 (5.2-7.9)	106	2.5 (1.7-3.8)	51	0.9 (0.6-1.4)	60	1.6 (0.9-2.6)	65	1.2 (0.9-1.7)	31	0.6 (0.4-0.9)	24	0.4 (0.3-0.7)	19	0.5 (0.3-0.9)	17	0.3 (0.2-0.6)	12	0.2 (0.1-0.4)
χ^2^ Value	1		48.9		59.3		16.3		17.5		33.2		72.6		16.8		32.6		4.6	
*P* value^c^	.38		.10		.13		.56		.36		.26		.006		.58		.24		.84	
**Insurance in pediatric population only**																				
Uninsured	11	4.9 (2.3-9.8)	1	0.4 (0.1-2.7)	5	2.3 (0.8-6.7)	1	0.4 (0.1-2.7)	1	0.3 (0.0-2.1)	1	0.6 (0.1-3.9)	0	0	1	0.2 (0.0-1.5)	1	0.3 (0.0-1.8)	0	0
Private insurance	104	5.7 (4.4-7.4)	29	1.5 (0.9-2.6)	26	1.6 (0.9-3.0)	9	0.4 (0.2-0.8)	20	0.8 (0.5-1.4)	10	0.4 (0.2-0.9)	6	0.3 (0.1-0.7)	13	0.8 (0.5-1.5)	5	0.4 (0.1-1.0)	2	0.1 (0.0-0.4)
Public insurance	35	7.0 (4.7-10.5)	3	0.2 (0.1-0.9)	12	3.2 (1.6-6.4)	7	1.1 (0.5-2.6)	2	0.2 (0.0-0.8)	3	1.3 (0.4-1.2)	1	0.1 (0.0-0.5)	2	0.3 (0.1-1.2)	0	0	2	0.3 (0.1-1.2)
χ^2^ Value	0.53		26.8		20.3		13.9		12.8		18		5.1		10.6		7.4		4.1	
*P* value^c^	.59		.01		.27		.15		.08		.26		.39		.14		.42		.56	
**Race and ethnicity in adult population only**																				
Asian	294	11.4 (9.8-13.3)	75	2.9 (2.0-4.2)	48	1.5 (1.0-2.0)	99	3.8 (3.0-4.9)	32	1.1 (0.8-1.7)	36	1.1 (0.7-1.7)	28	1.0 (0.6-1.5)	10	0.4 (0.2-0.7)	22	0.7 (0.5-1.2)	4	0.2 (0.1-0.5)
Black	663	11.2 (10.2-12.3)	144	2.4 (1.9-2.9)	137	2.3 (1.9-2.9)	205	3.3 (2.8-3.9)	93	1.6 (1.2-2.1)	61	1.0 (0.7-1.5)	55	0.9 (0.6-1.2)	33	0.6 (0.4-1.0)	38	0.5 (0.4-0.7)	16	0.2 (0.1-0.4)
Hispanic or Latino	875	11.6 (10.5-12.8)	230	2.4 (2.0-2.9)	173	2.6 (2.1-3.3)	274	3.4 (2.8-4.0)	120	1.5 (1.1-1.9)	80	1.2 (0.8-1.8)	109	1.5 (1.1-1.9)	60	0.8 (0.5-1.1)	67	1.0 (0.7-1.3)	29	0.3 (0.2-0.5)
White	4140	10.1 (9.7-10.6)	695	1.5 (1.4-1.7)	696	1.6 (1.5-1.8)	1077	2.6 (2.4-2.8)	473	1.1 (1.0-1.2)	293	0.6 (0.5-0.7)	336	0.7 (0.6-0.8)	319	0.9 (0.7-1.0)	228	0.5 (0.5-0.6)	95	0.2 (0.2-0.3)
Multiple or other^b^	322	15.9 (13.6-18.6)	40	1.2 (0.8-1.7)	71	3.7 (2.5-5.4)	77	4.3 (3.1-6.0)	26	0.8 (0.5-1.4)	20	0.8 (0.5-1.4)	23	1.0 (0.6-1.7)	19	0.7 (0.4-1.1)	21	1.0 (0.5-1.7)	5	0.2 (0.1-0.5)
χ^2^ Value	9.6		96.9		127.5		62.4		28.4		59.1		64.8		15.6		39		7.7	
*P* value^c^	<.001		<.001		<.001		<.001		.01		.001		<.001		.15		.002		.35	
**Annual household income in adult population only, $**																				
<25 000	1174	10.6 (9.8-11.5)	171	1.4 (1.1-1.7)	227	2.0 (1.6-2.4)	329	3.0 (2.5-3.5)	126	1.1 (0.9-1.4)	88	0.9 (0.6-1.2)	126	1.2 (0.9-1.5)	82	0.8 (0.6-1.1)	78	0.8 (0.6-1.1)	19	0.2 (0.1-0.3)
25 000 to 49 999	1694	10.9 (10.2-11.6)	301	1.6 (1.4-1.9)	304	1.9 (1.7-2.2)	451	2.8 (2.4-3.2)	204	1.2 (1.0-1.5)	126	0.8 (0.6-1.0)	135	0.8 (0.6-1.0)	110	0.7 (0.6-1.0)	91	0.6 (0.5-0.9)	30	0.2 (0.1-0.3)
50 000 to 99 999	2243	11.6 (9.6-12.3)	480	2.1 (1.9-2.4)	416	2.1 (1.9-2.4)	635	3.2 (2.9-3.6)	260	1.2 (1.1-1.5)	191	0.9 (0.7-1.1)	189	0.9 (0.7-1.0)	156	0.8 (0.7-1.1)	140	0.7 (0.6-0.9)	66	0.3 (0.2-0.4)
100 000 to 149 999	792	10.5 (9.6-11.5)	166	2.0 (1.7-2.5)	112	1.6 (1.2-2.1)	217	3.0 (2.5-3.6)	105	1.2 (1.0-1.5)	63	0.9 (0.6-1.4)	71	0.8 (0.6-1.1)	64	0.9 (0.6-1.2)	44	0.4 (0.3-0.6)	24	0.3 (0.2-0.5)
≥150 000	391	8.8 (7.7-10.0)	66	1.3 (1.0-1.7)	66	1.8 (1.3-2.5)	100	2.2 (1.7-2.8)	49	0.9 (0.7-1.3)	22	0.3 (0.2-0.5)	30	0.7 (0.4-1.0)	29	0.6 (0.4-0.9)	23	0.4 (0.2-0.6)	10	0.2 (0.1-0.3)
χ^2^ Value	4.5		46.5		13.9		26		7.2		29.2		19.4		7.2		26.9		6.9	
*P* value^c^	.001		<.001		.36		.07		.51		.05		.08		.61		.02		.50	
**Insurance in adult population only**																				
Uninsured	45	13.5 (9.3-19.3)	3	0.3 (0.1-0.9)	9	1.9 (0.9-3.8)	13	3.6 (1.8-7.1)	5	0.8 (0.3-2.3)	1	0.9 (0.1-6.0)	5	2.3 (0.8-6.3)	0	0	3	1.0 (0.2-3.9)	0	0
Private insurance	325	11.3 (9.9-12.9)	33	1.1 (0.7-1.6)	55	2.1 (1.5-3.0)	84	3.2 (2.5-4.2)	35	1.2 (0.8-1.8)	16	0.4 (0.2-0.7)	12	0.4 (0.2-0.7)	25	1.0 (0.6-1.6)	18	0.6 (0.3-1.1)	4	0.2 (0.1-0.8)
Public insurance	184	12.4 (10.4-14.8)	10	1.0 (0.4-2.2)	33	1.9 (1.2-2.8)	44	3.2 (2.2-4.5)	14	0.9 (0.4-1.9)	9	0.4 (0.2-0.9)	18	1.2 (0.7-1.9)	16	1.1 (0.5-2.4)	8	0.6 (0.3-1.4)	1	0 (0.0-0.3)
χ^2^ Value	0.62		3.9		0.7		0.4		2.6		3		34.2		6.9		1.3		5	
*P* value^c^	.53		.38		.83		.94		.55		.57		.003		.37		.79		.38	

^a^ Sample size (No.) presented is unweighted. Point estimates are weighted to reflect the national population.

^b^ Other race category includes those who self-reported their race as other, American Indian or Alaska Native, or Native Hawaiian or Other Pacific Islander.

^c^ Significant at *P* < .05.

### Convincing FA Prevalence by Socioeconomic Factors

Significant differences in the prevalence of any convincing FA were observed by household income. Convincing FA was most prevalent among households earning $50 000 to $99 999 per year (10.7% [95% CI, 10.2%-11.3%]) and lowest among those earning $150 000 or more (8.3% [95% CI, 7.4%-9.2%]) (Table 2). No significant differences in the prevalence of convincing FA were observed by insurance type.

### Multiple FAs, Severity, and Reaction Management

Among those with FAs, Black individuals had the highest rate of multiple convincing FAs (50.6% [95% CI, 46.1%-55.1%]) compared with other races and ethnicities (Table 4). Among respondents with convincing FAs, a history of at least 1 severe convincing FA reaction was also highest among Black individuals (55.8% [95% CI, 51.7%-59.8%]), followed by Hispanic individuals (51.3% [95% CI, 47.0%-55.5%]). Asian and White individuals had the lowest rates of severe food allergy reactions (Asian individuals, 46.9% [95% CI, 39.8%-54.1%] and White individuals, 47.8% [95% CI, 45.9%-49.7%]) compared with individuals of other races and ethnicities. Hispanic and Black individuals had higher rates of FA-related ED visits in the last year (Hispanic, 15.5% [95% CI, 12.3%-19.5%]; Black, 13.5% [95% CI, 9.7%-18.4%]) as well as in their lifetime (Hispanic, 47.7% [95% CI, 43.5%-52.0%]; Black, 45.4% [95% CI, 40.8%-50.1%]) compared with other races and ethnicities. In addition, rates of epinephrine autoinjector (EAI) use were highest among Black and Hispanic individuals (Asian, 22.6% [95% CI, 17.7%-28.%%]; Black, 23.6% [95% CI, 20.3%-27.2%]; Hispanic, 24.6% [95% CI, 21.7%-27.9%]; White, 20.9% [95% CI, 19.5%-22.4%]; >1 or other race, 19.4% [14.7%-25.3%]), but no significant differences in overall rates of EAI use (*P* = .14), or presence of a current EAI prescription (Asian, 28.0% [95% CI, 22.4%-34.4%]; Black: 26.7% [95% CI, 23.3%-30.5%]; Hispanic: 30.4% [95% CI, 27.1%-34.1%]; White, 26.0% [95% CI, 24.4%-27.6%]; >1 or other race, 23.6% [18.5%-29.7%]; *P* = .11) were observed by race and ethnicity.

**Table 4.** Frequency of Food Allergy Characteristics by Race, Ethnicity, Income, and Insurance

Characteristic	Severe		EAI prescription		ED visit in last year		Lifetime ED visit		Multiple food allergies		EAI use	
	No.^a^	% (95% CI)	No.^a^	% (95% CI)	No.^a^	% (95% CI)	No.^a^	% (95% CI)	No.^a^	% (95% CI)	No.^a^	% (95% CI)
**Race and ethnicity**												
Asian	188	46.9 (39.8-54.1)	136	28.0 (22.4-34.4)	52	9.9 (7.1-13.8)	153	36.5 (29.7-43.8)	180	37.2 (31.3-43.6)	101	22.6 (17.7-28.5)
Black	557	55.8 (51.7-59.8)	341	26.7 (23.3-30.5)	137	13.5 (9.7-18.4)	472	45.4 (40.8-50.1)	517	50.6 (46.1-55.1)	275	23.6 (20.3-27.2)
Hispanic	702	51.3 (47.0-55.5)	519	30.4 (27.1-34.1)	204	15.5 (12.3-19.5)	678	47.7 (43.5-52.0)	662	45.4 (41.2-49.6)	416	24.6 (21.7-27.9)
White	2969	47.8 (45.9-49.7)	2048	26.0 (24.4-27.6)	705	8.3 (7.5-9.3)	2445	35.6 (33.8-37.4)	2716	43.8 (41.9-45.6)	1564	20.9 (19.5-22.4)
Multiple or other^b^	306	50.7 (43.4-57.8)	182	23.6 (18.5-29.7)	72	8.4 (5.9-11.9)	229	33.9 (28.1-40.2)	265	39.4 (33.2-46.0)	132	19.4 (14.7-25.3)
χ^2^ Value	2.8		1.9		8		10.9		3.5		1.7	
*P* value^c^	.03		.11		<.001		<.001		.01		.14	
**Annual household income, $**												
<25 000	806	54.0 (50.3-57.6)	382	20.9 (18.4-23.8)	205	16.2 (12.9-20.0)	644	44.3 (40.6-48.2)	694	43.8 (40.2-47.4)	323	19.3 (16.7-22.3)
25 000-49 999	1205	50.0 (47.1-52.9)	667	22.3 (20.1-24.7)	240	8.9 (7.3-10.7)	990	37.7 (34.9-40.5)	1081	44.0 (41.2-46.9)	528	18.1 (16.1-20.2)
50 000-99 999	1774	49.3 (46.7-51.8)	1386	29.7 (27.6-32.0)	481	10.5 (8.7-12.6)	1586	40.9 (38.4-43.6)	1650	46.6 (44.0-49.3)	1060	25.2 (23.1-27.3)
100 000-149 999	629	47.7 (43.6-51.9)	526	31.0 (27.5-34.8)	163	8.3 (6.3-10.9)	511	35.1 (31.3-39.1)	604	42.4 (38.4-46.5)	389	21.9 (19.1-25.0)
≥150 000	308	45.9 (40.1-51.9)	265	28.9 (24.5-33.8)	81	7.7 (5.8-10.2)	246	33.6 (28.5-39.1)	311	42.5 (37.2-48.1)	188	24.2 (19.9-29.2)
χ^2^ Value	1.88		9.4		7.1		4.7		1.1		5.4	
*P* value^c^	.11		<.001		<.001		.001		.35		<.001	
**Insurance status**												
Uninsured	31	54.7 (38.9-69.5)	7	15.2 (6.6-31.1)	3	3.7 (1.1-11.4)	22	39.2 (24.7-55.9)	23	34.5 (22.1-49.4)	14	22. 3 (11.9-38.1)
Private insurance	195	47.1 (41.0-53.3)	89	22.2 (17.3-28.0)	28	8.0 (4.9-12.8)	109	26.5 (21.4-32.2)	189	41.2 (35.5-47.2)	61	14.0 (10.3-18.7)
Public insurance	110	49.0 (41.0-57.0)	27	7.8 (5.0-12.2)	28	16.7 (9.0-28.8)	73	35.6 (26.5-46.0)	109	50.8 (41.5-60.1)	28	9.5 (6.1-14.4)
χ^2^ Value	0.4		7.2		3.7		2.1		2.3		2.5	
*P* value^c^	.67		.001		.04		.12		.10		.08	

Abbreviations: EAI, epinephrine autoinjector; ED, emergency department.

^a^ Sample size (No.) presented is unweighted. Point estimates are weighted to reflect the national population.

^b^ Other race category includes those who self-reported their race as other, American Indian or Alaska Native, or Native Hawaiian or Other Pacific Islander.

^c^ Significant at *P* < .05.

Patient report of a severe FA reaction history was more common among lower earning households, but again this difference was not statistically significant. In contrast, differences in rates of current epinephrine prescriptions were significantly different by household income, with the lowest earning households least likely to report a current EAI prescription. Report of at least 1 FA-related ED visit (in the last year and lifetime) was most frequent among those with a household income less than $25 000 (last year, 16.2% [95% CI, 12.9%-20.0%]; lifetime, 44.3% [95% CI, 40.6%-48.2%]) (Table 4).

When observing FA severity by insurance type, no significant differences were observed except for the rate of EAI prescription (*P* < .001), which was the highest for those with private insurance (22.2% [95% CI, 17.3%-28.0%]), as well as rates of FA-related ED visits in the past year, which were most common among publicly-insured respondents (16.7% [95% CI, 9.0%-28.8%]) (Table 4).

### Associations Between Race and Ethnicity With Convincing FA

Odds ratios were generated from a model of logistic regressions adjusted for sex, age, geographic region, household income, and atopic comorbidities. Compared with White individuals, Asian individuals (adjusted odds ratio [AOR], 1.21 [95% CI, 1.02-1.43]; *P* = .03), Black individuals (AOR, 1.15 [95% CI, 1.03-1.29]; *P* = .02), Hispanic individuals (AOR, 1.17 [95% CI, 1.04-1.30]; *P* = .006), and those categorized as having more than 1 race or other races (AOR, 1.46 [95% CI, 1.23-1.71]; *P* < .001) were more likely to have at least 1 convincing FA (Figure).

**Figure.**
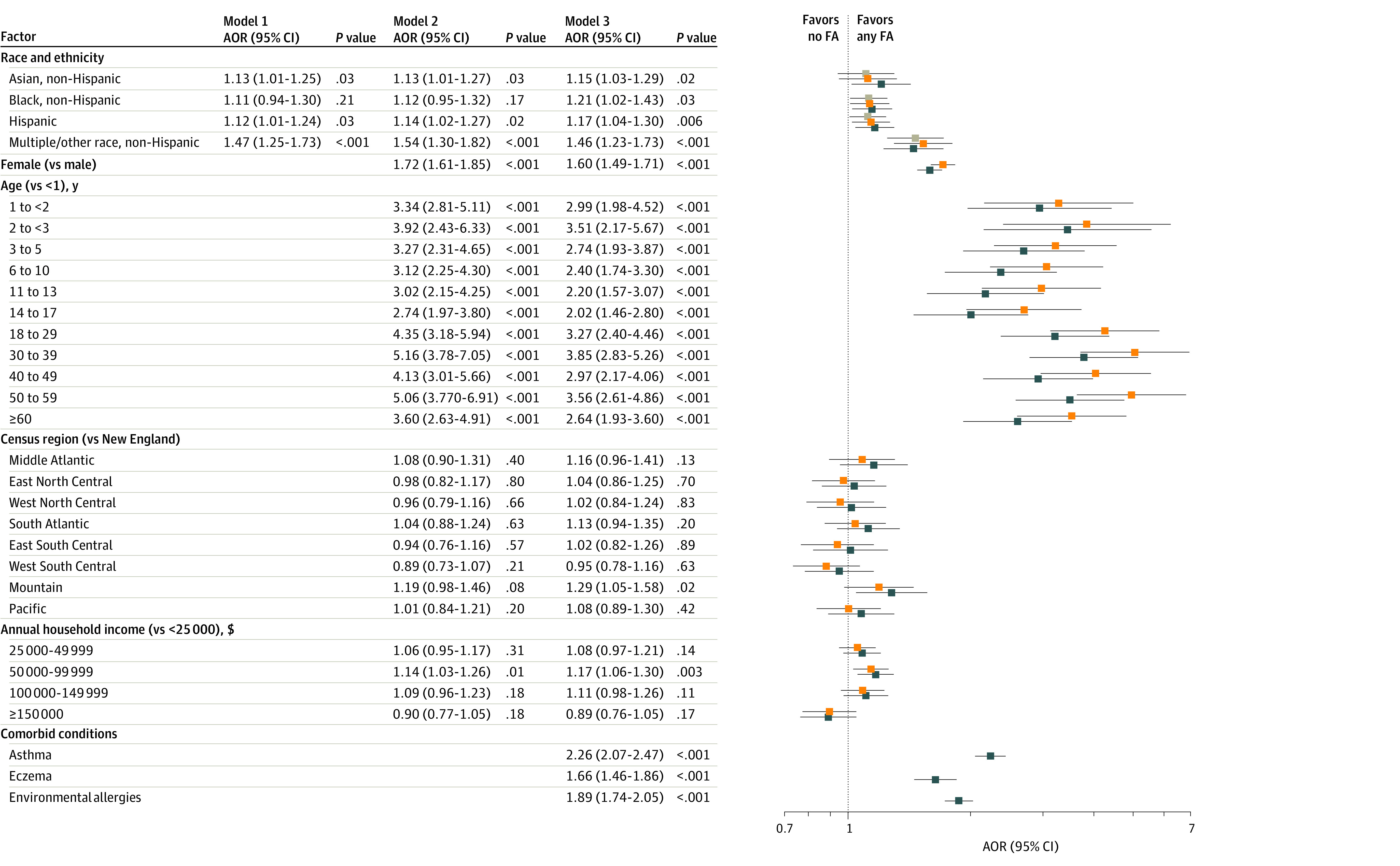
Factors Associated With Any Convincing Food Allergy (FA) The different colors reflect estimates from models with different covariate sets (gray, model 1; orange, model 2; blue, model 3). Estimates with the same color are from the same model. AOR indicates adjusted odds ratio.

## Discussion

To our knowledge, this is one of the few studies to estimate the prevalence of convincing FAs among children and adults living in the US using a national, probability-based sampling frame and a diverse sample with respect to race, ethnicity, and socioeconomic status. White individuals across all ages had lower rates of convincing FAs (9.5%) compared with Asian, Black, and Hispanic individuals (Asian, 10.5%; Black, 10.6%; Hispanic, 10.6%). Specific food allergen types and rates of FA outcomes (severe allergic reactions, multiple allergies, and ED visits) systematically varied across individuals of different racial and ethnic backgrounds. In addition, a convincing FA and a history of severe FA were seemingly lower among high-income families. This study demonstrates that FA burden disproportionately falls on individuals who report a non-White race and ethnicity^19^ as well as individuals with lower household incomes.

Asian children reported the highest rates of tree nut allergy, and Black children reported the highest rate of egg allergy and fin fish allergy. In addition, Asian adults reported the highest rate of peanut allergy and shellfish allergy, Black adults reported the highest rate of tree nut allergy, and Hispanic adults reported the highest rate of hen’s egg allergy and fin fish allergy. These findings corroborate existing literature that suggests specific food allergens are more common among different racial and ethnic groups.^15^ Data collected over a decade ago suggested that Black children and adults exhibited elevated shellfish allergy risk relative to White children and adults.^15^ More recent literature from the FORWARD study demonstrated that shellfish allergy and fin fish allergy were more common among Black children than White children.^20,21^ Findings from our study suggest that seafood allergy is also more common among Asian and Hispanic populations. It is unclear what factors are associated with seafood allergy, but previous literature has hypothesized that it may be mediated by differential sensitization to household-level environmental exposures, such as dust mites or cockroaches.^22,23^ These exposures may be present as a result of environmental injustices latently established through historically racially, ethnically, and socioeconomically biased policies.^24^ Separately, in an online survey of South Asian Indian individuals living in the US, tree nut allergy was reported to be the most common FA.^25^ Previous studies from Australia have also demonstrated that tree nut allergies were disproportionately prevalent among children living in Australia who were born to Asian parents.^26^ Our study sample included Asian individuals from all regions of origin. Considering the heterogeneity within racial and ethnic groups with respect to dietary practices, environmental exposures, and genetic ancestry, as well as the paucity of literature investigating the distribution of FAs among racially and ethnically diverse adult populations, future FA studies should consider further assessment of sociocultural and economic characteristics and explore associations with FA outcomes among individuals of different racial and ethnic backgrounds.

Black and Hispanic individuals reported higher rates of severe allergic reactions, allergies to multiple foods, and FA-related ED visits compared with those from other racial and ethnic groups. These national data build on previous published analyses of clinical data from tertiary care medical centers in the Midwestern US, which reported that Black and Hispanic patients with FAs may experience more FA burden compared with patients from other racial or ethnic backgrounds.^4^ Previous data from Florida demonstrated that Black and Hispanic children had higher rates of food-induced anaphylaxis and ED visits.^27^ In an observational study of ED data from New York and Florida, ED rates for food-induced anaphylaxis were highest among young Black children living in urban environments.^28^ In a secondary analysis of the US National Mortality Database from 1999 to 2010, rates of fatal food-induced anaphylaxis significantly increased among African American male patients but not among other demographic groups.^14^ A better understanding of how socioeconomic factors and barriers are associated with FA outcomes is necessary to develop effective interventions to improve FA management. Considering the disproportionate FA burden among Black and Hispanic individuals in the US, as well as those from households with lower incomes, further research is necessary to explore barriers and facilitators to FA management that are specifically experienced by these individuals and families to allow for the development of more targeted, culturally relevant, equitable, and accessible educational efforts that will improve FA outcomes and eliminate FA-related disparities for these understudied and historically marginalized groups.

The prevalence of convincing FAs and the frequency of a history of severe FA reactions were lowest among those with a household income of $150 000 or more. However, paradoxically, among the few studies focused on household income and FA prevalence, previous data have suggested that those in lower income brackets have lower FA prevalence. A National Center for Health Statistics data brief reported that FA prevalence increased as income level increased from 1997 to 2011.^29^ The prevalence of FA was reported in reference to the national poverty level at the time of analysis: less than 100% of the poverty level, between 100% and 200%, and above 200%, which differ from the 5 categories used for the analyses of our study. In addition, only 0.6% of children enrolled in Medicaid had an FA diagnosis^30^ compared with the general population of children with a confirmed, physician-diagnosed FA prevalence rate of 5%.^1^ However, children and adults enrolled in Medicaid may not be able to obtain an accurate physician diagnosis of FA because there is less access to specialist care. Many private practices do not accept Medicaid coverage,^31^ and academic centers that accept it are concentrated in urban areas, a challenge for individuals living in suburban or rural areas. The limited access to care may inflate self-reported or parent-reported FAs because there is a barrier to obtaining clinical assessments and diagnostic testing, such as skin prick tests, specific IgE assessments, and oral food challenges, to identify cases of FA. Fewer reports of severe FA reactions among individuals with higher household incomes may be associated with having more access to FA management. Individuals using government-sponsored nutritional support programs have difficulty accessing allergen-free options.^32^ Only 1 in 2 children with an FA enrolled in Medicaid have a filled EAI prescription.^33^ Although further research is necessary to better understand the potential association of household income with FA outcomes, these findings emphasize the importance of socioeconomic factors as likely associated with FA outcomes by race and ethnicity. These socioeconomic factors should be factored into future analyses of the association of race and ethnicity with FA because these social constructs may influence each other.

### Limitations

This study has some limitations. Although this study used US Census racial and ethnic categories, each category represents a heterogeneous population, of which we did not individually analyze subpopulations. Limitations also exist in the collapsed classification of multiracial individuals and other races. Due to the limited sample size of multiracial individuals, individuals of other races, Native Hawaiian or Other Pacific Islander individuals, and American Indian or Alaska Native individuals, they were categorized as individuals of other races for the purposes of these analyses. In addition, our findings were limited by their reliance on self-reported or parent-reported data. Self-reported or parent-reported data are subject to recall bias and often overpresent FA cases because individuals may mistakenly include intolerances and oral allergy syndrome as an FA. It is not as accurate as the criterion standard oral food challenge used to identify a true clinical FA. However, oral food challenges are not feasible to estimate FA prevalence on a national level because they are costly and time-consuming. Recognizing the potential clinical underrepresentation and overestimation of FA using self-reported or parent-reported data, this study implemented a strict, convincing FA definition considering symptoms and food allergens to reasonably estimate FA prevalence. Finally, the survey was conducted only in English and Spanish, which may have led to underrepresentation of Asian populations and other immigrant populations with limited English- or Spanish-language fluency.

## Conclusion

This survey study suggests that, in the US, Asian, Black, and Hispanic populations appear to experience greater FA burden compared with their White counterparts. Further efforts should be undertaken to evaluate the sociocultural and economic covariates associated with racial and ethnic differences in FA burden and to explore additional factors such as cultural heterogeneity within racial and ethnic groups experiencing FAs. Additional targeted, educational interventions may address disparities in FA outcomes and improve targeted FA management.

## Notes

Supplement 1. **eFigure.** Prevalence of Convincing and Physician-Confirmed Food Allergy by Age, Estimated Across Racial, Ethnic, and Household Income Strata

Supplement 2.
Data Sharing Statement

## References

[1] Gupta RS, Warren CM, Smith BM, . The public health impact of parent-reported childhood food allergies in the United States. Pediatrics. 2018;142(6):e20181235. doi:10.1542/peds.2018-1235 30455345 PMC6317772

[2] Gupta RS, Warren CM, Smith BM, . Prevalence and severity of food allergies among US adults. JAMA Netw Open. 2019;2(1):e185630. doi:10.1001/jamanetworkopen.2018.5630 30646188 PMC6324316

[3] Warren CM, Jiang J, Gupta RS. Epidemiology and burden of food allergy. Curr Allergy Asthma Rep. 2020;20(2):6. doi:10.1007/s11882-020-0898-7 32067114 PMC7883751

[4] Mahdavinia M, Fox SR, Smith BM, . Racial differences in food allergy phenotype and health care utilization among US children. J Allergy Clin Immunol Pract. 2017;5(2):352-357. doi:10.1016/j.jaip.2016.10.006 27888035 PMC8215518

[5] Warren C, Bartell T, Nimmagadda SR, Bilaver LA, Koplin J, Gupta RS. Socioeconomic determinants of food allergy burden: a clinical introduction. Ann Allergy Asthma Immunol. 2022;129(4):407-416. doi:10.1016/j.anai.2022.07.021 35914663

[6] Keet CA, Savage JH, Seopaul S, Peng RD, Wood RA, Matsui EC. Temporal trends and racial/ethnic disparity in self-reported pediatric food allergy in the United States. Ann Allergy Asthma Immunol. 2014;112(3):222-229.e3. doi:10.1016/j.anai.2013.12.007 24428971 PMC3950907

[7] Gupta RS, Springston EE, Warrier MR, . The prevalence, severity, and distribution of childhood food allergy in the United States. Pediatrics. 2011;128(1):e9-e17. doi:10.1542/peds.2011-0204 21690110

[8] Greenhawt M, Weiss C, Conte ML, Doucet M, Engler A, Camargo CA Jr. Racial and ethnic disparity in food allergy in the United States: a systematic review. J Allergy Clin Immunol Pract. 2013;1(4):378-386. doi:10.1016/j.jaip.2013.04.009 24565543

[9] McGowan EC, Keet CA. Prevalence of self-reported food allergy in the National Health and Nutrition Examination Survey (NHANES) 2007-2010. J Allergy Clin Immunol. 2013;132(5):1216-1219.e5. doi:10.1016/j.jaci.2013.07.018 23992749 PMC3822433

[10] Keet CA, Wood RA, Matsui EC. Personal and parental nativity as risk factors for food sensitization. J Allergy Clin Immunol. 2012;129(1):169-75.e1, 5. doi:10.1016/j.jaci.2011.10.002 22075329 PMC3382052

[11] Joseph CLM, Zoratti EM, Ownby DR, . Exploring racial differences in IgE-mediated food allergy in the WHEALS birth cohort. Ann Allergy Asthma Immunol. 2016;116(3):219-224. doi:10.1016/j.anai.2015.12.019 26837607 PMC4864956

[12] Liu AH, Jaramillo R, Sicherer SH, . National prevalence and risk factors for food allergy and relationship to asthma: results from the National Health and Nutrition Examination Survey 2005-2006. J Allergy Clin Immunol. 2010;126(4):798-806.e13. doi:10.1016/j.jaci.2010.07.026 20920770 PMC2990684

[13] Kumar R, Tsai HJ, Hong X, . Race, ancestry, and development of food-allergen sensitization in early childhood. Pediatrics. 2011;128(4):e821-e829. doi:10.1542/peds.2011-0691 21890831 PMC3182844

[14] Jerschow E, Lin RY, Scaperotti MM, McGinn AP. Fatal anaphylaxis in the United States, 1999-2010: temporal patterns and demographic associations. J Allergy Clin Immunol. 2014;134(6):1318-1328. doi:10.1016/j.jaci.2014.08.018 25280385 PMC4260987

[15] Sicherer SH, Muñoz-Furlong A, Sampson HA. Prevalence of seafood allergy in the United States determined by a random telephone survey. J Allergy Clin Immunol. 2004;114(1):159-165. doi:10.1016/j.jaci.2004.04.018 15241360

[16] Vierk KA, Koehler KM, Fein SB, Street DA. Prevalence of self-reported food allergy in American adults and use of food labels. J Allergy Clin Immunol. 2007;119(6):1504-1510. doi:10.1016/j.jaci.2007.03.011 17451802

[17] Ford ME, Kelly PA. Conceptualizing and categorizing race and ethnicity in health services research. Health Serv Res. 2005;40(5, pt 2):1658-1675. doi:10.1111/j.1475-6773.2005.00449.x 16179001 PMC1361221

[18] US Census Bureau. 2020 Census frequently asked questions about race and ethnicity. Accessed May 11, 2023. https://www.census.gov/programs-surveys/decennial-census/decade/2020/planning-management/release/faqs-race-ethnicity.html

[19] Martinez A, de la Rosa R, Mujahid M, Thakur N. Structural racism and its pathways to asthma and atopic dermatitis. J Allergy Clin Immunol. 2021;148(5):1112-1120. doi:10.1016/j.jaci.2021.09.020 34743832 PMC9186508

[20] Branum AM, Lukacs SL. Food allergy among children in the United States. Pediatrics. 2009;124(6):1549-1555. doi:10.1542/peds.2009-1210 19917585

[21] Thivalapill N, Andy-Nweye AB, Bilaver LA, ; of the FORWARD team. Sensitization to house dust mite and cockroach may mediate the racial difference in shellfish allergy. Pediatr Allergy Immunol. 2022;33(8):e13837. doi:10.1111/pai.13837 36003047 PMC12341321

[22] Wong L, Huang CH, Lee BW. Shellfish and house dust mite allergies: is the link tropomyosin? Allergy Asthma Immunol Res. 2016;8(2):101-106. doi:10.4168/aair.2016.8.2.101 26739402 PMC4713872

[23] McGowan EC, Peng R, Salo PM, Zeldin DC, Keet CA. Cockroach, dust mite, and shrimp sensitization correlations in the National Health and Nutrition Examination Survey. Ann Allergy Asthma Immunol. 2019;122(5):536-538. doi:10.1016/j.anai.2019.02.015 30802502 PMC6500746

[24] Cook Q, Argenio K, Lovinsky-Desir S. The impact of environmental injustice and social determinants of health on the role of air pollution in asthma and allergic disease in the United States. J Allergy Clin Immunol. 2021;148(5):1089-1101. doi:10.1016/j.jaci.2021.09.018 34743831

[25] Jiang J, Dinakar C, Fierstein JL, Gupta OK, Gupta RS. Food allergy among Asian Indian immigrants in the United States. J Allergy Clin Immunol Pract. 2020;8(5):1740-1742. doi:10.1016/j.jaip.2019.12.026 31917364

[26] Wang Y, Allen KJ, Suaini NHA, Peters RL, Ponsonby AL, Koplin JJ. Asian children living in Australia have a different profile of allergy and anaphylaxis than Australian-born children: a state-wide survey. Clin Exp Allergy. 2018;48(10):1317-1324. doi:10.1111/cea.13235 30025179

[27] Harduar-Morano L, Simon MR, Watkins S, Blackmore C. A population-based epidemiologic study of emergency department visits for anaphylaxis in Florida. J Allergy Clin Immunol. 2011;128(3):594-600. doi:10.1016/j.jaci.2011.04.049 21714994 PMC3970843

[28] Sakai-Bizmark R, Friedlander SMI, Oshima K, . Urban/rural residence effect on emergency department visits arising from food-induced anaphylaxis. Allergol Int. 2019;68(3):316-320. doi:10.1016/j.alit.2018.12.007 30737115

[29] Jackson KD, Howie LD, Akinbami LJ. Trends in allergic conditions among children: United States, 1997-2011. NCHS Data Brief. 2013;(121):1-8. 23742874

[30] Bilaver LA, Kanaley MK, Fierstein JL, Gupta RS. Prevalence and correlates of food allergy among Medicaid-enrolled United States children. Acad Pediatr. 2021;21(1):84-92. doi:10.1016/j.acap.2020.03.005 32200110

[31] Hsiang WR, Lukasiewicz A, Gentry M, . Medicaid patients have greater difficulty scheduling health care appointments compared with private insurance patients: a meta-analysis. Inquiry. 2019;56. doi:10.1177/0046958019838118 30947608 PMC6452575

[32] Frame A, Katari P, Wang J, Bagley S, Cook Q. Impact of access to allergen-free food options through WIC on food allergy-related quality of life. Ann Allergy Asthma Immunol. 2022;129(5):S64. doi:10.1016/j.anai.2022.08.685

[33] Kanaley MK, Dyer AA, Negris OR, . Guideline-informed care among Medicaid-enrolled children with food allergy. Am J Manag Care. 2020;26(12):505-512. doi:10.37765/ajmc.2020.88538 33315325

