# Supplementary material for: Racial, Ethnic, and Socioeconomic Differences in Food Allergies in the US
Source: JAMA Netw Open. 2023 Jun 14;6(6):e2318162. doi: 10.1001/jamanetworkopen.2023.18162 (PMC10267771; doi:10.1001/jamanetworkopen.2023.18162)
Supplement: Supplement 1. — eFigure. Prevalence of Convincing and Physician-Confirmed Food Allergy by Age, Estimated Across Racial, Ethnic, and Household Income Strata [file jamanetwopen-e2318162-s001.pdf]

Supplementary Online Content

Jiang J, Warren CM, Brewer A, Soffer G, Gupta RS. Racial, ethnic, and socioeconomic differences in food allergies in the US. *JAMA Netw Open.* 2023;6(6):e2318162. doi:10.1001/jamanetworkopen.2023.18162

eFigure. Prevalence of Convincing and Physician-Confirmed Food Allergy by Age, Estimated Across Racial, Ethnic, and Household Income Strata

This supplementary material has been provided by the authors to give readers additional information about their work.

© 2023 Jiang J et al. *JAMA Network Open.*

eFigure. Prevalence of Convincing and Physician-Confirmed Food Allergy by Age, Estimated Across Racial, Ethnic, and Household Income Strata

Prevalence of Any Convincing FA By Age and Race/Ethnicity

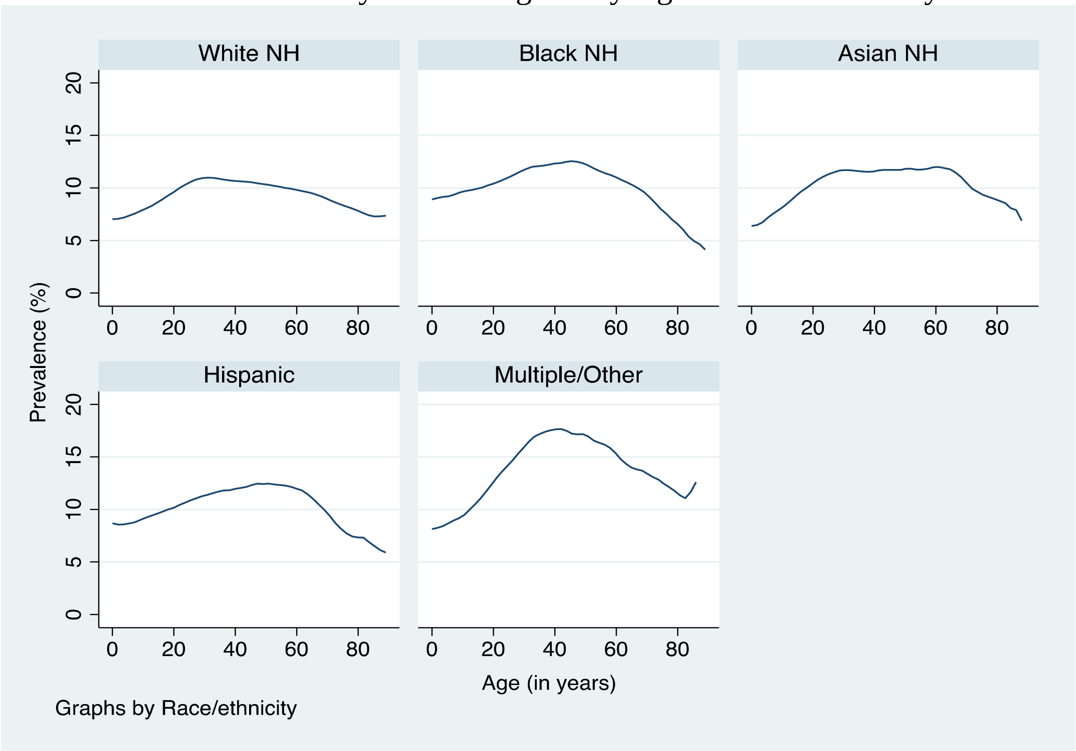

Prevalence of Any Confirmed FA By Age and Race/Ethnicity

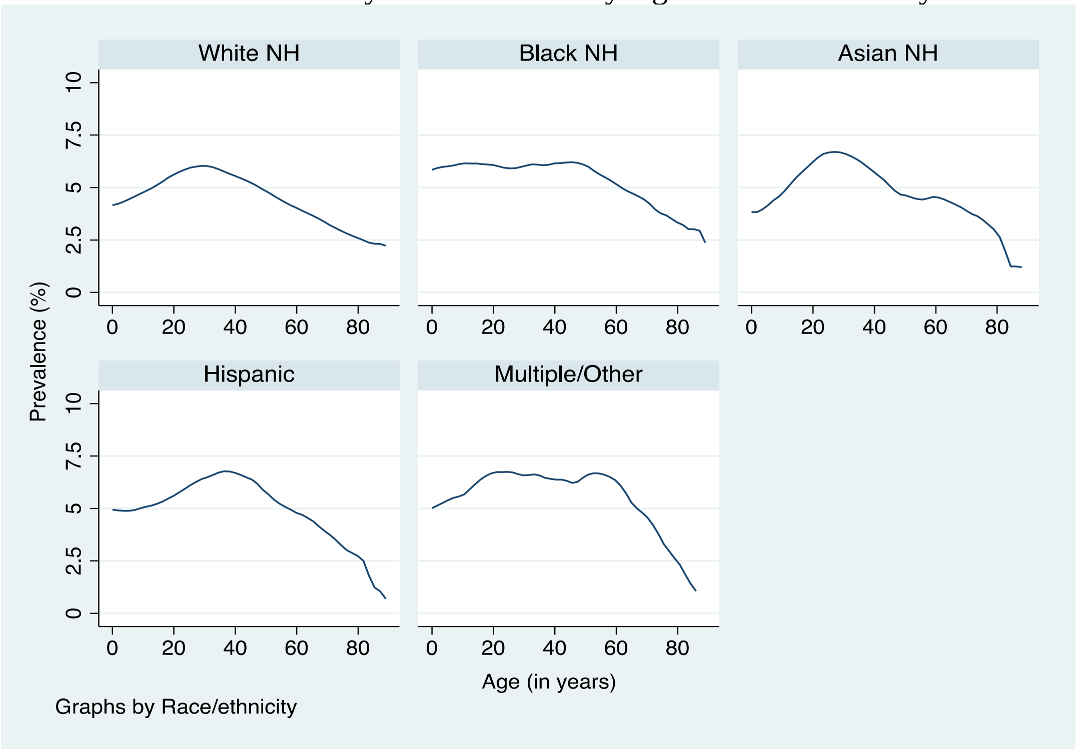

© 2023 Jiang J et al. *JAMA Network Open.*

Prevalence of Any Convincing FA By Age and Annual Household Income

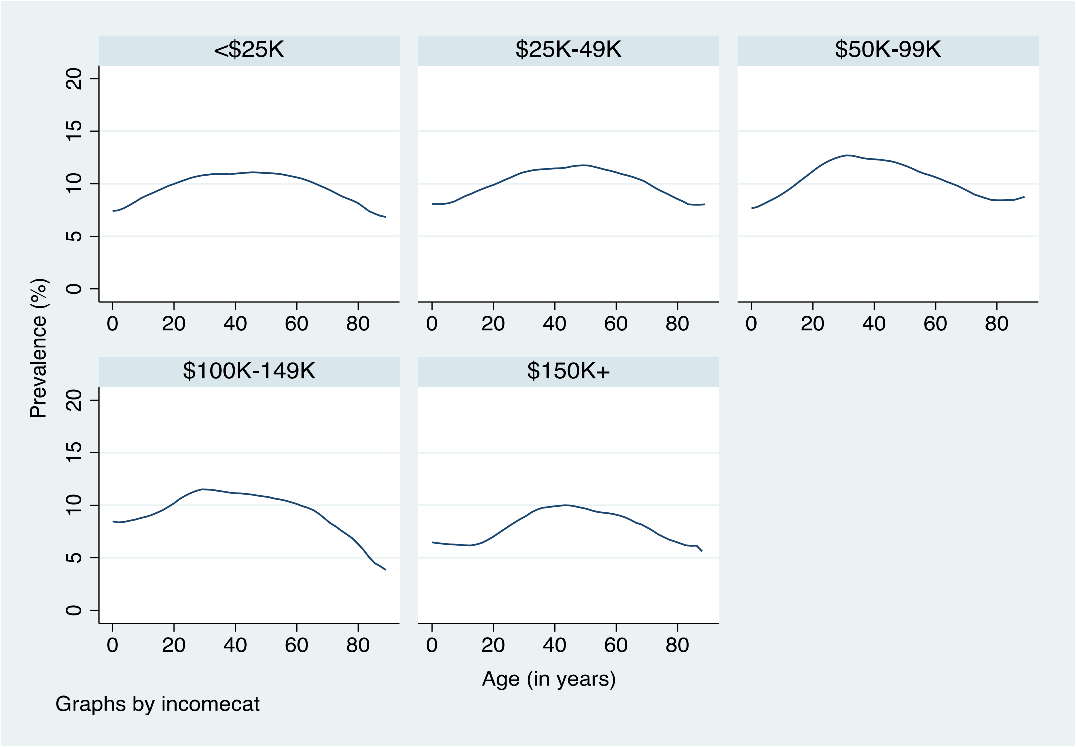

Prevalence of Any Confirmed FA By Age and Annual Household Income

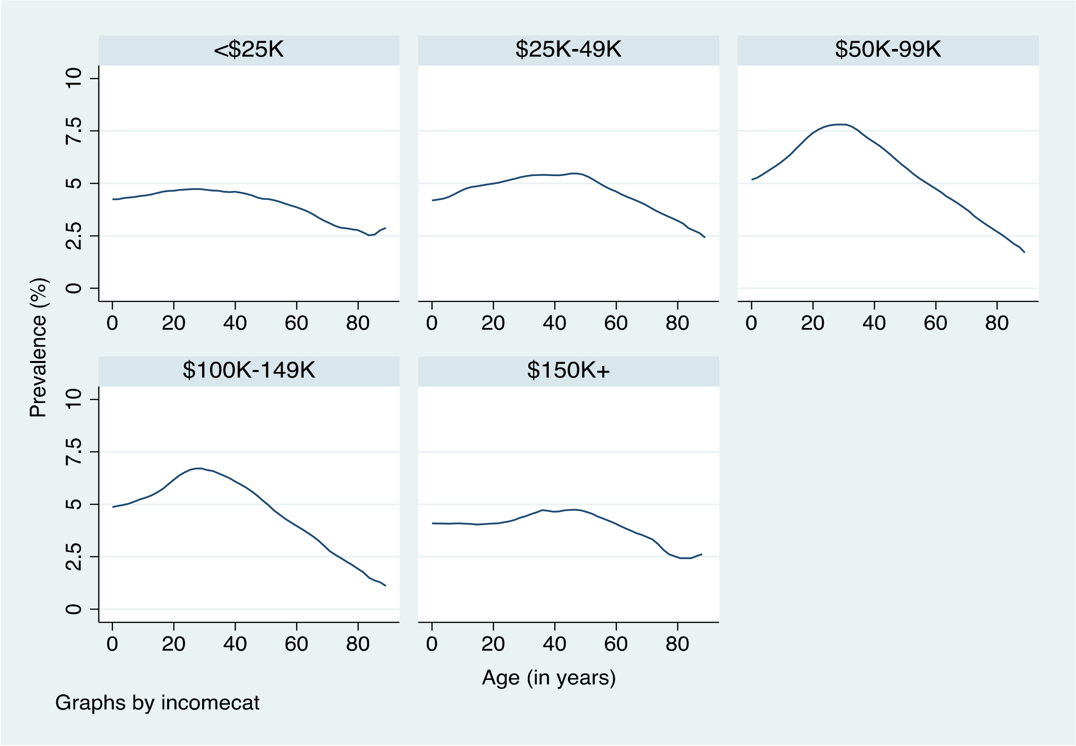

© 2023 Jiang J et al. *JAMA Network Open.*
