# Supplementary material for: Racial, Ethnic, and Socioeconomic Differences in Food Allergies in the US
Source: JAMA Netw Open. 2023 Jun 14;6(6):e2318162. doi: 10.1001/jamanetworkopen.2023.18162 (PMC10267771; doi:10.1001/jamanetworkopen.2023.18162)
Supplement: Supplement 2. — Data Sharing Statement [file jamanetwopen-e2318162-s002.pdf]

Data Sharing Statement

Jiang. Racial, Ethnic, and Socioeconomic Differences in Food Allergies in the US. *JAMA Netw Open*. Published June 14, 2023. doi:10.1001/jamanetworkopen.2023.18162

Data
Data available: No
